# ICTV virus taxonomy profile: *Picobirnaviridae*

**DOI:** 10.1099/jgv.0.001186

**Published:** 2018-11-28

**Authors:** Bernard Delmas, Houssam Attoui, Souvik Ghosh, Yashpal S. Malik, Egbert Mundt, Vikram N. Vakharia, ICTV Report Consortium

**Affiliations:** ^1^​ VIM, INRA, Université Paris-Saclay, 78350, Jouy-en-Josas, France; ^2^​ UMR1161 Virologie, ANSES, INRA, Ecole Nationale Vétérinaire d'Alfort, Université Paris-Est, Maisons-Alfort, France; ^3^​ Department of Biomedical Sciences, Ross University School of Veterinary Medicine, St.Kitts and Nevis; ^4^​ Indian Veterinary Research Institute, Izatnagar 243 122, Uttar Pradesh, India; ^5^​ Boehringer Ingelheim, Ingelheim am Rhein, Germany; ^6^​ Department of Marine Biotechnology, University of Maryland, Baltimore County, 701, EastPratt Street, Baltimore, MD 21202, USA

**Keywords:** *Picobirnaviridae*, ICTV Report, taxonomy

## Abstract

*Picobirnaviridae* is a family of viruses with bi-segmented (rarely unsegmented) dsRNA genomes comprising about 4.4 kbp in total, with small, non-enveloped spherical virions. The family includes one genus (*Picobirnavirus*) grouping three genetic clusters with high sequence variability, two defined by viruses infecting vertebrates and a third with viruses found in invertebrates. This is a summary of the International Committee on Taxonomy of Viruses (ICTV) Report on the taxonomy of *Picobirnaviridae*, which is available at www.ictv.global/report/picobirnaviridae.

## VIRION

**Fig. 1.**
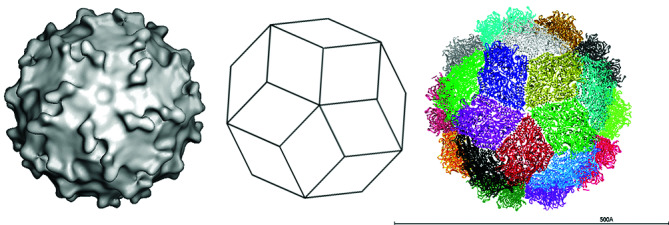
Structure of a picobirnavirus particle. (Left) Surface rendering from a three-dimensional reconstruction viewed down a slightly mis-oriented fivefold axis. Note the presence of 60 dimeric protrusions (courtesy of J. Lepault). (Middle) Diagram representing a triacontahedron, a convex polyhedron comprising 30 rhombic faces or diamond tiles. (Right) Triacontahedral design of the particle, with each of the tiles formed by two capsid protein dimers coloured differently (courtesy of S. Duquerroy).

Virus particles are isometric, non-enveloped and 33–37 nm in diameter. Recombinant virion-like particles have a spherical triacontahedral (30-sided) organization (Table 1, Fig. 1), with a unique layer of 60 symmetric capsid protein dimers [1].

**Table 1.** Characteristics of the family *Picobirnaviridae*

Typical member: human picobirnavirus, Hy005102 (RNA1: AB186897; RNA2: AB186898), species *Human picobirnavirus*, genus *Picobirnavirus*	
Virion	Non-enveloped, spherical virion, 33–37 nm in diameter
Genome	Two double-stranded RNA segments, each of 1.7–2.7 kbp
Replication	Not known due to lack of infection models in cell culture or animals
Translation	Not known
Host range	Vertebrates and invertebrates
Taxonomy	One genus

## GENOME

**Fig. 2.**
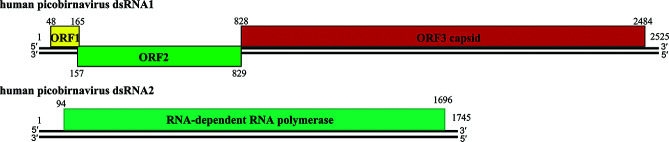
Schematic representation of the gene arrangement in genome dsRNA1 and dsRNA2 of human picobirnavirus strain Hy005102. Numbers indicate nucleotide positions.

Most picobirnavirus genomes consist of two linear dsRNA segments. Segment 2 (dsRNA2, 1.7–1.9 kbp) encodes an RNA-dependent RNA-polymerase, while the larger segment 1 (dsRNA1, 2.4–2.7 kbp) has two large open reading frames (ORF2 and ORF3) that can be preceded by a small ORF (ORF1) [2] (Fig. 2). The functions of ORF 1 and ORF2 are unknown, but ORF3 encodes a capsid protein precursor which is auto-catalytically cleaved 50–70 residues from its N-terminus to generate a positively charged peptide and mature capsid protein [1]. The 5′-(GUAAA) and 3′-(ACUGC) terminal nucleotide sequences are conserved in segment 2 of picobirnaviruses from various host species [2, 3]. The consensus bacterial ribosomal binding site sequence (AGGAGG) occurs in most picobirnavirus 5′-untranslated regions.

Monosegmented picobirnavirus genomes that result from a fusion of genomic segments 1 and 2, the RNA-dependent RNA polymerase gene being located at the 3′-end, have been detected in vertebrates and invertebrates [4, 5].

## REPLICATION

The RNA-dependent RNA polymerase is active with single-strand RNA and double-stranded RNA templates and transcription proceeds in a semi-conservative manner. The RNA-dependent RNA polymerase cannot be incorporated into recombinant capsids in the absence of the viral genome [6] .

## PATHOGENESIS

Picobirnaviruses have been associated with diarrhoea in immunocompromised individuals [7] and children, but have also been detected in stool samples and the respiratory tract of individuals and animals without gastroenteritis [8]. Picobirnaviruses can persistently infect pigs [9].

## TAXONOMY

Two species in the single genus *Picobirnavirus* are *Human picobirnavirus* and *Rabbit picobirnavirus*. Phylogenetic analysis of picobirnavirus complete RNA-dependent RNA polymerase and capsid protein sequences isolated from vertebrates (*n*=20) and invertebrates (*n*=7) reveals the presence of three genogroups. Genogroups I and II are limited to vertebrate viruses and are represented by the reference strain 1-CHN-97 for genogroup I (this genogroup includes human picobirnavirus, Hy005102, a member of the species *Human picobirnavirus*) and 4-GA-91 for genogroup II. Genogroup III consists of viruses derived from invertebrates, and these are also distinguished in that their RNA-dependent RNA polymerases lack the ExxRxNxxxE motif typical of vertebrate picobirnaviruses. The divergent viruses constituting each of these genogroups may comprise multiple species.

Segments encoding a picobirna-like RNA-dependent RNA polymerase using an alternative mitochondrial genetic code have also been identified [5, 10].

Picobirnavirus genome organization and content are similar to those of partitiviruses.

## RESOURCES

Full ICTV Report: www.ictv.global/report/picobirnaviridae.
